# Molecular Heterogeneity Within Normozoospermia: A Pilot Pooled Small RNA Sequencing Study

**DOI:** 10.3390/biom16101514

**Published:** 2026-10-18

**Authors:** Maria-Anna Kyrgiafini, Aris Kaltsas, Georgios Stamatellos, Georgia Vetsika, Zissis Mamuris

**Affiliations:** 1 Laboratory of Genetics, Comparative and Evolutionary Biology, Department of Biochemistry and Biotechnology, University of Thessaly, Viopolis, Mezourlo, 41500 Larissa, Greece; mkyrgiafini@uth.gr (M.-A.K.); stamatellosgeo@gmail.com (G.S.); gvetsik@gmail.com (G.V.); 2 Third Department of Urology, Attikon University Hospital, School of Medicine, National and Kapodistrian University of Athens, 12462 Athens, Greece; ares-kaltsas@hotmail.com

**Keywords:** male fertility, reproduction, noncoding RNA (ncRNA), spermatozoa, sperm miRNA

## Abstract

Normozoospermia, defined by semen parameters above the WHO lower reference limits, is conventionally treated as a single category. Increasing evidence suggests that this classification may encompass molecular heterogeneity. This pilot-pooled sequencing study investigated whether men occupying distinct phenotypes within the normozoospermic range exhibit different sperm miRNA profiles. Thirty normozoospermic men were stratified into three investigator-defined groups based on sperm concentration and normal morphology: an intermediate concentration/morphology group (NORMAV), a higher-concentration group (>80 × 10^6^/mL; NORMCOUNT), and a higher-morphology group (>9% normal forms; NORMMOR). Sperm small RNA sequencing was performed using two biological replicate pools per group, followed by differential expression (*q-value* < 0.01, |log_2_FC| > 1) and functional analyses restricted to strongly validated miRNA–target interactions. Pool-level differences in miRNA abundance were identified across all pairwise comparisons, with miR-34c-3p, miR-891b, miR-145-5p, and miR-1-3p among the recurrently dysregulated miRNAs. Functional enrichment of database-curated miRNA targets suggested convergence on proliferation, apoptosis, development, and intracellular signaling, including the PI3K–Akt, MAPK, and FoxO pathways. 568 genes were shared across all comparisons, with *VEGFA*, *IGF1R*, and *PTEN* among the most recurrent targets. These exploratory findings indicate pool-level sperm miRNA variation among the investigated normozoospermic groups and identify candidate miRNAs for subsequent individual-level validation. Larger studies using individual-sample sequencing and orthogonal validation are required to determine whether these differences are consistently observed across participants.

## 1. Introduction

Infertility is a major reproductive health problem worldwide, with the World Health Organization (WHO) estimating a lifetime prevalence of approximately 17.5% and a period prevalence of 12.6% among adults for 1990–2021 [1]. Male-related factors contribute to nearly half of all infertility cases, either independently or in combination with female factors [2], highlighting the importance of accurate assessment of male reproductive health. Male infertility is a complex and multifactorial condition involving genetic, anatomical, and endocrine factors, while environmental and lifestyle exposures may further impair male reproductive function through endocrine disruption and other interconnected mechanisms, ultimately affecting spermatogenesis, sperm quality, and reproductive potential [3,4]. Despite considerable advancements in reproductive medicine and assisted reproductive technologies, the accurate characterization of male fertility potential remains elusive, necessitating a more comprehensive understanding of the biological and molecular mechanisms that govern male reproductive function.

Conventional semen analysis remains the cornerstone of male fertility assessment, providing quantitative and qualitative information on parameters such as semen volume, sperm concentration, motility, and morphology [5,6]. Men whose semen characteristics lie above the lower reference limits of the WHO laboratory manual are conventionally described as normozoospermic; these limits, however, describe reference distributions and are not thresholds separating fertile from infertile men [6]. Normozoospermia does not necessarily represent a biologically uniform condition, as substantial variation in sperm concentration, total sperm count, progressive motility, morphology, vitality and total motile sperm count may exist among men who satisfy the WHO reference criteria [7]. Consequently, two individuals categorized as normozoospermic may exhibit substantially different positions within the normal reference ranges, although whether different positions within these continuous distributions correspond to stable differences in spermatogenic efficiency, endocrine regulation, sperm maturation, cellular function, or molecular integrity remains uncertain [7,8,9]. Furthermore, semen characteristics are continuous quantitative traits subject to both inter- and intra-individual variability, influenced by factors such as abstinence duration, age, febrile illness, lifestyle, medication, oxidative stress, environmental exposures, and natural fluctuations in spermatogenesis [10,11]. Therefore, the dichotomous classification of semen profiles as normal or abnormal may obscure biologically significant variation among men within the same diagnostic category. This limitation is particularly pertinent in cases of unexplained infertility, where men may exhibit apparently normal conventional semen parameters despite impaired reproductive outcomes [12,13,14]. Molecular differences in sperm composition, functional capacity, and regulatory mechanisms may thus remain undetected by routine semen analysis, highlighting the potential value of molecular characterization for elucidating biological heterogeneity within the normozoospermic population.

Mature spermatozoa are highly specialized cells that undergo extensive structural and molecular remodeling during spermatogenesis and epididymal maturation. Traditionally considered primarily as carriers of the paternal genome, spermatozoa are now recognized to harbor a complex RNA cargo. This cargo comprises both coding and non-coding RNA species, including messenger RNAs (mRNAs), long non-coding RNAs (lncRNAs), circular RNAs (circRNAs), PIWI-interacting RNAs (piRNAs), transfer RNA-derived small RNAs (tsRNAs), and microRNAs (miRNAs) [15,16,17,18]. Among these, miRNAs are short, single-stranded non-coding RNAs, approximately 18–24 nucleotides in length, that regulate gene expression predominantly at the post-transcriptional level through sequence-specific interactions with target mRNAs, leading to translational repression and/or transcript degradation [19]. During male germ cell development, miRNAs participate in the regulation of key processes involved in spermatogenesis, including germ cell proliferation and differentiation, meiotic progression, and sperm maturation [20,21]. Mature spermatozoa, which are largely transcriptionally inactive, retain a distinct repertoire of miRNAs that reflects, at least in part, molecular events occurring during spermatogenesis and epididymal maturation rather than ongoing transcription [21]. Moreover, sperm-borne miRNAs are delivered to the oocyte at fertilization; causal effects on early post-fertilization events and embryonic development have been demonstrated predominantly in animal models, whereas human evidence remains largely associative [22,23,24]. Accordingly, the presence or differential abundance of a given miRNA in mature human sperm should not itself be interpreted as evidence of a functional role in fertilization or embryonic development. Importantly, altered sperm miRNA expression profiles have been associated with impaired semen quality and various male infertility phenotypes, suggesting that sperm miRNAs may provide molecular information beyond that captured by conventional semen parameters [21,25]. Collectively, these findings support the investigation of sperm miRNA signatures as a means of further characterizing the molecular complexity underlying male reproductive phenotypes.

Despite increasing evidence supporting the involvement of sperm miRNAs in male reproductive function, most studies have predominantly focused on comparisons between infertile men or individuals with impaired semen parameters and normozoospermic subjects serving as reference groups [20,21,26]. Such study designs frequently assume normozoospermia to be a relatively homogeneous reference phenotype, even though considerable variation in sperm concentration, motility, and morphology exist among individuals who satisfy the WHO reference criteria [7]. Emerging evidence, however, indicates the presence of molecular heterogeneity even among men who share a normozoospermic phenotype. Notably, Salas-Huetos et al. (2016) demonstrated that normozoospermic fertile and infertile men exhibited distinct sperm miRNA expression profiles, despite the absence of conventional seminal abnormalities in the infertile group [9]. Similarly, Denomme et al. (2017) reported altered sperm DNA methylation patterns and miRNA profiles in normozoospermic men associated with poor blastocyst development, further indicating that molecular alterations with potential reproductive relevance may exist despite conventionally normal semen parameters [27]. More recently, sperm RNA profiling in normozoospermic men undergoing fertility treatment identified hundreds of RNA elements associated with blastocyst progression, further supporting the existence of molecular variation within conventionally normal semen phenotypes [28]. In addition to reproductive outcome, paternal age has also been associated with differences in the sperm miRNA profiles of normozoospermic men, even in the absence of substantial alterations in conventional semen parameters [29]. Collectively, these findings suggest that molecular variation may exist among normozoospermic men that is not captured by routine semen analysis. Nevertheless, studies specifically investigating molecular variation among distinct semen phenotypes within the normozoospermic range remain limited.

In this context, the present pilot pooled-sequencing study aimed to investigate molecular heterogeneity within normozoospermia by characterizing sperm miRNA expression profiles among investigator-defined normozoospermic phenotypic groups defined by sperm concentration and morphology. Using small RNA sequencing, we compared an investigator-defined intermediate normozoospermic group with groups characterized by markedly high sperm concentration or a high proportion of morphologically normal spermatozoa. The grouping strategy was intended to compare selected concentration- and morphology-based profiles within the normozoospermic range rather than to isolate the independent molecular effects of sperm concentration or morphology. Differentially expressed miRNAs were identified across these phenotypic groups, followed by target gene identification and functional enrichment analyses to explore their potential biological relevance. The novelty of this exploratory analysis lies in the specific comparison of investigator-defined concentration- and morphology-based strata within the normozoospermic range. The aim of this exploratory analysis was to generate hypotheses regarding candidate pool-level miRNA differences among these predefined groups rather than to establish individual-level molecular signatures or predict reproductive potential.

## 2. Materials and Methods

### 2.1. Study Population and Sample Collection

This exploratory pilot pooled-sequencing study enrolled thirty (*n* = 30) normozoospermic men aged 27–49 years. Eligible participants were healthy adult men whose semen parameters were above the lower reference limits of the WHO laboratory manual (6th edition); these limits were used operationally for eligibility and were not interpreted as boundaries between fertility and infertility Exclusion criteria included reproductive tract disorders (such as varicocele, cryptorchidism, orchitis, epididymitis, or genital tract infections), endocrine disorders affecting male reproduction, chromosomal abnormalities, Y chromosome microdeletions, active malignancy, systemic inflammatory or autoimmune diseases, or any other condition known to impair spermatogenesis. Men receiving medications known to affect spermatogenesis, as well as those with excessive alcohol consumption, illicit drug use, or recent febrile illness, were also excluded.

All participants underwent a comprehensive andrological evaluation, encompassing medical history assessment, physical examination, and scrotal ultrasonography when clinically indicated. In addition, participants completed a detailed questionnaire eliciting information on their medical and reproductive history, lifestyle habits, and other pertinent clinical data.

Semen samples were collected via masturbation following 2–5 days of sexual abstinence. Semen analysis was conducted in an accredited andrology laboratory in accordance with the WHO Laboratory Manual for the Examination and Processing of Human Semen (6th edition). Routine semen parameters, including semen volume, sperm concentration, total sperm count, progressive and total motility, and sperm morphology, were evaluated according to standardized WHO recommendations. Cell-Vision counting slides (Tek-Event, Sydney, Australia) were used for sperm counting, and microscopic evaluation was performed using Nikon Eclipse TS100, Nikon Eclipse E200, and Nikon Eclipse Ts2 microscopes (Nikon, Tokyo, Japan). Two independent ejaculates were collected from each participant approximately 3–5 months apart; both samples were used for subsequent RNA isolation and contributed to the RNA pooling procedure. Two independent semen analyses were performed on separate ejaculates collected from each participant. Phenotypic classification required fulfillment of the predefined group-specific numerical criteria in both assessments; therefore, only participants classified into the same phenotypic group in both analyses were included.

Based on the results of two independent semen analyses, participants were stratified into three mutually exclusive phenotypic groups (*n* = 10 per group). All participants fulfilled the WHO 6th-edition reference criteria for normozoospermia in both assessments. The NORMAV group comprised men with sperm concentrations of 30–75 × 10^6^ spermatozoa/mL and normal morphology ≥ 4% but <9% in both analyses. The NORMCOUNT group comprised men with sperm concentrations > 80 × 10^6^ spermatozoa/mL and normal morphology < 9% in both analyses, whereas the NORMMOR group comprised men with normal morphology > 9% and sperm concentrations < 80 × 10^6^ spermatozoa/mL in both analyses. The selected cut-offs were investigator-defined exploratory thresholds intended to capture contrasting phenotypic profiles within the normozoospermic range while ensuring that all participants remained above the WHO lower reference limits. No participant simultaneously fulfilled the predefined criteria for the NORMCOUNT and NORMMOR groups. Only individuals who consistently fulfilled the respective group criteria across both semen assessments were included in the final analysis. The phenotypic stratification was specifically based on sperm concentration and normal morphology and was not intended to encompass the full spectrum of semen phenotypes defined by all conventional semen parameters. In particular, the >80 × 10^6^/mL sperm concentration criterion was used operationally to define a group with consistently higher sperm concentration than the intermediate-concentration NORMAV group and should not be interpreted as a clinical cutoff for high sperm concentration. The grouping strategy was not designed to isolate the independent effect of sperm concentration or morphology, nor to represent holistic semen phenotypes.

For the descriptive and between-group analyses of participant characteristics, the mean of the two semen assessments was calculated for each individual and used as the participant-level value. Continuous variables are presented as mean ± standard deviation (SD). Between-group differences were evaluated using Welch’s one-way analysis of variance (ANOVA), while the Kruskal–Wallis test was used for abstinence duration. Categorical variables, including smoking status and alcohol consumption, were compared using the chi-square test. A *p-value* < 0.05 was considered statistically significant.

The study protocol received approval from the Ethics Committee of the University of Thessaly (Volos, Greece), and all participants provided written informed consent prior to enrolment.

### 2.2. RNA Extraction and Sample Preparation

Prior to RNA extraction, sperm samples underwent a somatic cell lysis procedure to eliminate contaminating epithelial cells, leukocytes, and other non-sperm cells. Briefly, sperm pellets were incubated on ice for 20–30 min in a somatic cell lysis buffer containing 0.1% sodium dodecyl sulfate (SDS) and 0.5% Triton X-100 prepared in DEPC-treated water. Following centrifugation, the purified sperm pellets were washed twice with phosphate-buffered saline (PBS) to remove residual lysis buffer and cellular debris. Following somatic-cell lysis and washing, sperm preparations were examined microscopically to assess the removal of contaminating somatic cells. No additional assessment using somatic-cell- or leukocyte-specific molecular markers was performed.

Total RNA was subsequently extracted from the purified spermatozoa using the miRNeasy Mini Kit (Qiagen, Hilden, Germany), according to the manufacturer’s instructions. RNA concentration was determined using a Qubit 2.0 Fluorometer (Thermo Fisher Scientific, Waltham, MA, USA) in conjunction with the Qubit microRNA Assay Kit (Thermo Fisher Scientific, Waltham, MA, USA). RNA concentrations were >20 ng/μL for all samples, with a mean concentration of approximately 22 ng/μL. RNA purity was assessed using a NanoDrop 2000 spectrophotometer (Thermo Fisher Scientific, Waltham, MA, USA), with OD260/280 ratios of approximately 2.0. RNA integrity was additionally assessed by agarose gel electrophoresis prior to downstream library preparation. Following processing, samples were stored at −80 °C. Extracted RNA was maintained at −80 °C and sent for library preparation and sequencing within one month of RNA isolation.

A biological pooling strategy was implemented to obtain group-level expression profiles. Within each phenotypic group (*n* = 10), RNA samples were divided into two independent pools, each consisting of five individuals, yielding a total of six RNA pools. For each pool, equal amounts of RNA from each of the five individuals were combined to ensure equimolar representation of all samples. These two pools served as biological replicates for downstream small RNA sequencing and differential expression analyses. Within each phenotypic group, the ten participants were allocated into two independent biological replicate pools of five individuals each. Pool assignment was performed with the aim of generating two broadly comparable replicate pools with respect to the available demographic, lifestyle, and semen characteristics, while preserving the predefined phenotypic characteristics of each group. No formal individual matching was performed across corresponding pools of the three phenotypic groups. The individual composition and characteristics of each pool are provided in Supplementary Table S1. Individuals assigned to the same pool were selected to ensure comparable demographic and clinical characteristics, including age, lifestyle habits, and other relevant clinical variables, thereby minimizing the impact of potential confounding factors on small RNA expression profiles.

### 2.3. Small RNA Library Construction and Sequencing

Purified RNA samples were shipped on dry ice to Novogene Co. (Cambridge, UK) for small RNA library preparation and sequencing. Libraries were constructed in accordance with the provider’s standard small RNA library preparation protocol. Briefly, 3′ and 5′ adapters were sequentially ligated to the purified small RNA molecules, followed by first-strand cDNA synthesis using a reverse transcription primer. The resulting cDNA was PCR-amplified to generate double-stranded sequencing libraries, which were subsequently purified and size-selected to enrich fragments corresponding to small RNAs (18–40 nt).

Library quality was rigorously assessed by the sequencing provider prior to sequencing. The libraries were sequenced on an Illumina NovaSeq platform (Illumina, San Diego, CA, USA) utilizing a single-end 50 bp (SE50) sequencing strategy, yielding approximately 20 million raw reads per library.

All six small RNA libraries were prepared within the same library-preparation batch and sequenced in the same sequencing lane. Library order was randomized during processing to minimize systematic technical variation associated with sample order.

### 2.4. Bioinformatics Analysis

Raw sequencing data underwent an initial quality control process utilizing Trimmomatic v0.36 [30] to eliminate adapter-containing reads, low-quality reads, and sequences shorter than 18 nucleotides. The resulting high-quality reads were subsequently aligned to the human reference genome (GRCh38/hg38) using Bowtie 2 [31,32]. Known miRNAs were identified by alignment against the miRBase v20.0 [33] reference database, while sRNAtoolbox 2.0 [34] was employed for miRNA annotation and characterization. To minimize false-positive miRNA identification, reads were further compared against the RepeatMasker (http://www.repeatmasker.org) and Rfam [35] databases to identify repetitive sequences and other classes of non-coding RNAs, including ribosomal RNAs (rRNAs), transfer RNAs (tRNAs), small nucleolar RNAs (snoRNAs), small nuclear RNAs (snRNAs), and small cytoplasmic RNAs (scRNAs). Reads assigned to these RNA classes were excluded from subsequent miRNA identification and expression analyses. Novel miRNAs were predicted by integrating miREvo v1.1 [36] and mirDeep2 v2.0.1.2 [37], which evaluate precursor secondary structures, Dicer cleavage sites, and minimum free energy of unannotated small RNA reads. To ensure unambiguous annotation, each read was assigned to a single RNA category according to a predefined annotation hierarchy, prioritizing known miRNAs, followed by other annotated non-coding RNA species (rRNA, tRNA, snRNA, and snoRNA), repetitive sequences, genomic regions, and finally candidate novel miRNAs.

### 2.5. Analysis of Differentially Expressed miRNAs and Functional Enrichment of Their Target Genes

To descriptively assess global similarity among the six pooled small-RNA libraries, pairwise correlations of miRNA expression profiles were examined across all libraries, including both within-group and between-group comparisons. This analysis was used as an unsupervised descriptive assessment of the relationships among the six pooled expression profiles. Given the pooled design and the small number of libraries, the correlations were interpreted at the library level and not as evidence of individual-level reproducibility or phenotypic separation.

MiRNA expression abundance was quantified as transcripts per million (TPM) to account for differences in sequencing depth among libraries. Differential expression analysis was performed using raw miRNA read counts with the DESeq2 version 1.50.2 R/Bioconductor package [38]. Pairwise differential expression analyses were conducted for all group comparisons (NORMCOUNT vs. NORMAV, NORMAV vs. NORMMOR, and NORMCOUNT vs. NORMMOR). For each contrast, positive log_2_ fold-change values indicate higher miRNA abundance in the first group relative to the second group, whereas negative values indicate lower abundance in the first group relative to the second group.

The six pooled libraries were treated as independent experimental units, with two biological replicate pools representing each phenotypic group (NORMAV, NORMCOUNT, and NORMMOR). A DESeqDataSet was constructed using the design formula ~ group. Library-size normalization was performed using the DESeq2 median-of-ratios size-factor normalization procedure, followed by dispersion estimation and differential-expression testing within the negative-binomial generalized linear modeling framework. Pairwise contrasts were performed for NORMCOUNT vs. NORMAV, NORMAV vs. NORMMOR, and NORMCOUNT vs. NORMMOR. Raw *p-values* were corrected for multiple testing using the Storey *q-value* [39] approach for false discovery rate (FDR) estimation. miRNAs with a *q-value* < 0.01 and an absolute log_2_ fold change (|log_2_FC|) > 1 were considered differentially expressed. Given the limited number of pooled biological replicates, differential-expression results were interpreted as exploratory pool-level differences rather than estimates of individual-level miRNA variation. Extremely small *q-values* were reported as numerical zeros in the initial analysis output due to floating-point underflow, reflecting limitations in numerical precision rather than true *q-values* of zero. For visualization in the volcano plots, −log_10_(*q*) values exceeding 300 were capped at 300. This graphical capping was applied solely for visualization and did not affect the underlying statistical analysis or significance classification.

To explore biological processes and pathways associated with the differentially expressed miRNAs, their experimentally validated target genes were retrieved using miRTargetLink 2.0 [40]. Only experimentally validated miRNA–target interactions supported by strong evidence were included in the analysis. Novel candidate miRNAs were necessarily excluded from target retrieval, as no experimentally validated interactions exist for newly predicted miRNAs. For each comparison, only target genes shared by two or more differentially expressed miRNAs were retained for downstream functional enrichment analysis.

Gene Ontology (GO) enrichment analysis [41,42] and Kyoto Encyclopedia of Genes and Genomes (KEGG) pathway analysis [43] were performed using ShinyGO v0.85 [44] to identify significantly enriched biological processes and signaling pathways associated with the overlapping target genes. Statistical significance was assessed following false discovery rate (FDR) correction for multiple testing, with an FDR-adjusted *p-value* threshold of <0.05. Up- and downregulated miRNAs were analyzed jointly; consequently, no inference regarding the direction of pathway regulation was drawn, and enrichment results were interpreted as hypothesis-generating annotations rather than as evidence of pathway activation or inhibition.

**Figure 1:**
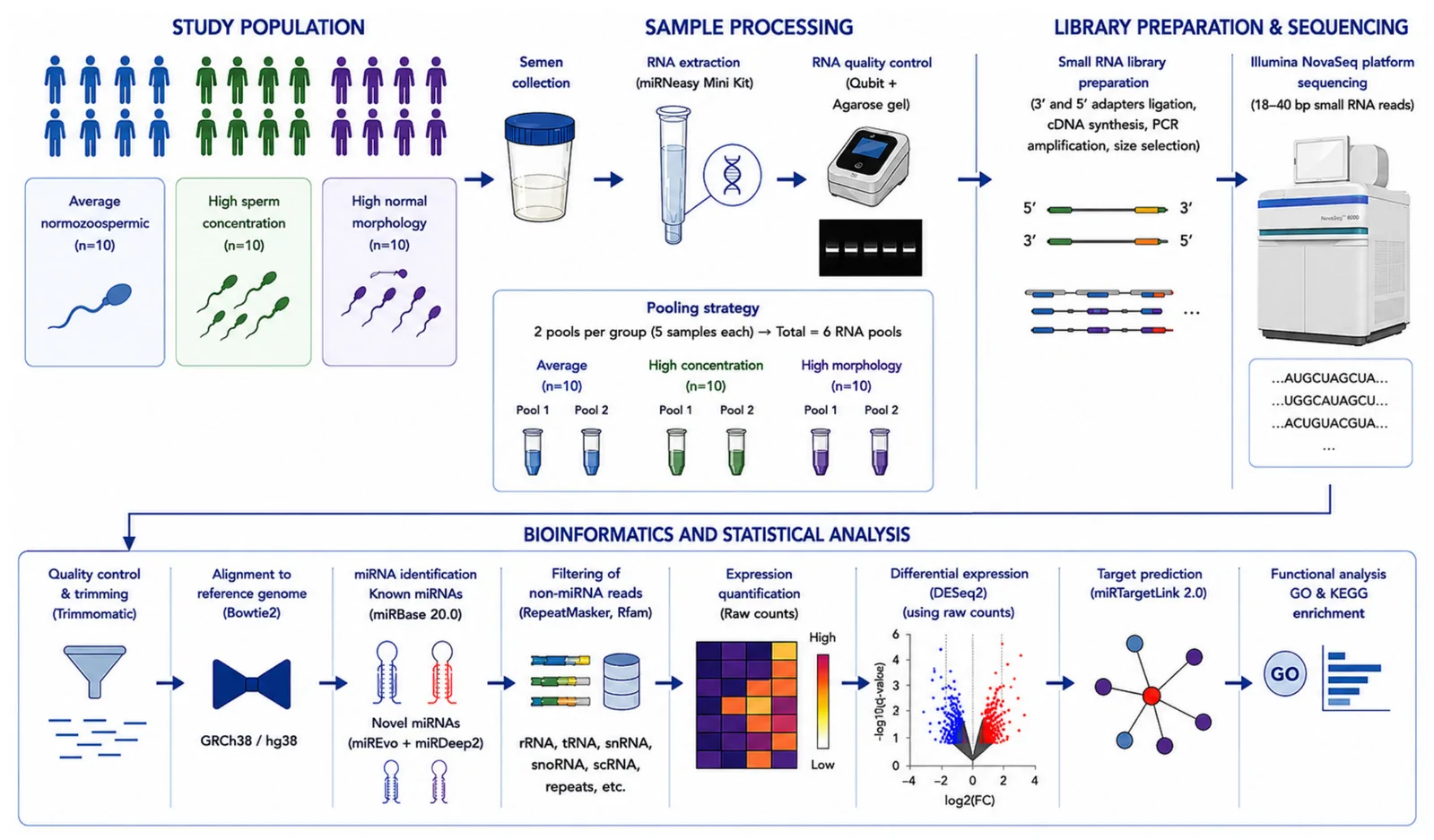
Overview of the experimental design and bioinformatics workflow used in the present study. Thirty normozoospermic men were stratified into three investigator-defined phenotypic groups based on sperm concentration and normal morphology, followed by sperm purification, RNA extraction, small RNA sequencing, differential expression analysis, retrieval of experimentally validated target genes, and functional enrichment analyses.

The overall experimental design, from participant stratification and sample processing to bioinformatics and functional enrichment analyses, is summarized in Figure 1.

## 3. Results

### 3.1. Participant and Semen Characteristics

Participant demographic, lifestyle, and semen characteristics are summarized in Table 1, while de-identified individual-level data from both semen assessments are provided in Supplementary Table S1. As expected from the predefined phenotypic stratification, significant between-group differences were observed for sperm concentration (*p* < 0.001) and normal morphology (*p* < 0.001). Total sperm count also differed significantly among the three groups (*p* < 0.001), consistent with the markedly higher sperm concentration characterizing the NORMCOUNT group. In contrast, no significant between-group differences were observed for age, abstinence period, BMI, semen volume, progressive motility, total motility, vitality, smoking status, or alcohol consumption (all *p* > 0.05). These findings indicate that, among the recorded characteristics, the groups were broadly comparable apart from the semen characteristics directly related to the predefined phenotypic stratification.

### 3.2. Small RNA Sequencing Data Quality and miRNA Identification

Small RNA sequencing generated between 20.43 and 21.48 million raw reads per library across the six biological replicates (Table 2). The total read count averaged 20,833,911, with individual sample values ranging from 20,430,697 (NORMAV1) to 21,482,577 (NORMCOUNT1). Following adapter removal and quality filtering, 19.49–20.89 million clean reads were retained, corresponding to 95.39–98.19% of the initial sequencing output. These consistently high read-retention rates indicated adequate sequencing quality across all libraries.

Alignment of the clean reads to the human reference genome (GRCh38/hg38) resulted in mapping rates ranging from 73.35% to 80.50%. The highest mapping rate was observed for NORMAV1 (80.50%), followed by NORMCOUNT1 (78.36%) and NORMAV2 (78.03%), while NORMMOR1 exhibited the lowest mapping rate (73.35%). Overall, sequencing depth, read-retention rates, and genome-mapping efficiency were consistent across the six libraries, thereby supporting the suitability of the generated datasets for subsequent miRNA identification and differential expression analyses.

Following sequence processing and genome alignment, known and novel miRNAs were identified across all six libraries (Table 3). The number of mapped known mature miRNAs ranged from 465 to 537, while 439–510 corresponding known precursor hairpin sequences were detected. NORMCOUNT libraries exhibited the highest numbers of both mature and precursor miRNAs, with 537 and 530 mature miRNAs and 510 and 506 hairpin sequences identified in NORMCOUNT1 and NORMCOUNT2, respectively. In comparison, the NORMAV libraries contained 465–469 mature miRNAs and 439–443 hairpin sequences, whereas 476–478 mature miRNAs and 453–454 hairpin sequences were identified in the NORMMOR libraries.

A smaller set of candidate novel miRNAs was also identified (Table 3). Across the six libraries, 12–15 novel mature miRNAs and 11–15 corresponding novel precursor hairpin sequences were detected. Similar to the known miRNA profiles, the highest numbers of novel miRNAs were observed in the NORMCOUNT libraries. Overall, the two biological replicates within each phenotypic group showed comparable numbers of identified known and novel miRNAs, indicating consistency in miRNA detection across replicate libraries.

**Figure 2:**
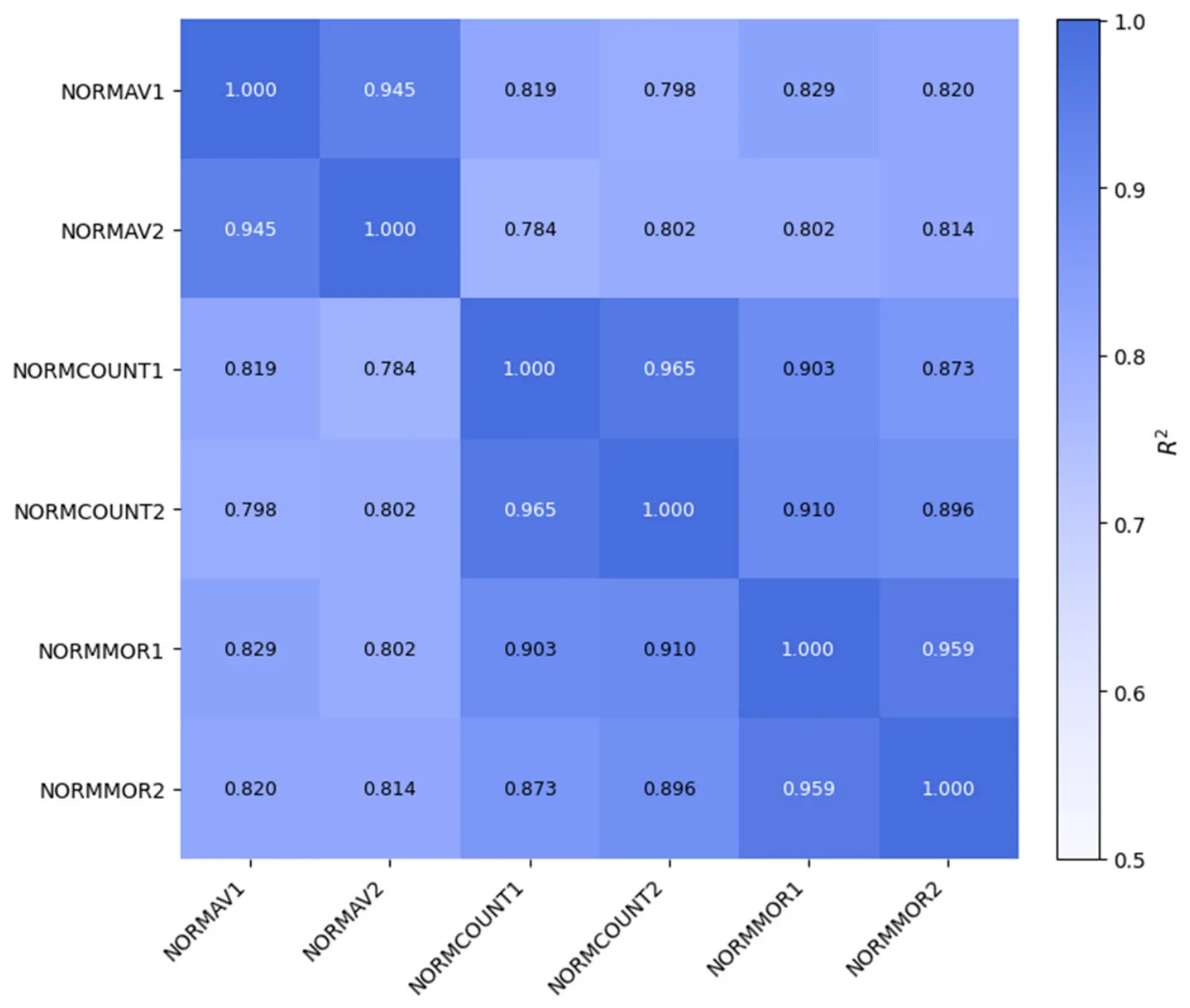
Correlation analysis of miRNA expression profiles across the six small RNA sequencing libraries. The heatmap displays the squared Pearson correlation coefficients (R^2^) derived from pairwise comparisons between the libraries of the NORMAV, NORMCOUNT, and NORMMOR groups. Higher R^2^ values indicate greater similarity between miRNA expression profiles.

Pearson correlation analysis was performed to assess the similarity of miRNA expression profiles among the six sequencing libraries (Figure 2), and the squared Pearson correlation coefficients (R^2^) were used for visualization and descriptive assessment. The resulting R^2^ values revealed high concordance between the two biological replicates within each normozoospermic group: 0.945 for NORMAV, 0.965 for NORMCOUNT, and 0.959 for NORMMOR. In comparison, R^2^ values between libraries from different phenotypic groups ranged from 0.784 to 0.910. NORMCOUNT and NORMMOR samples showed relatively higher inter-group similarity (R^2^ = 0.873–0.910), while comparisons involving NORMAV with either NORMCOUNT or NORMMOR generally exhibited lower values (R^2^ = 0.784–0.829). These R^2^ values were used descriptively to assess concordance between the two pooled libraries within each group. The consistently high R^2^ values indicated strong agreement between the pooled expression profiles; however, given the limited number of pooled libraries per group, they were not interpreted as evidence of individual-level reproducibility.

### 3.3. Differential miRNA Expression Among Normozoospermic Phenotypic Groups

**Figure 3:**
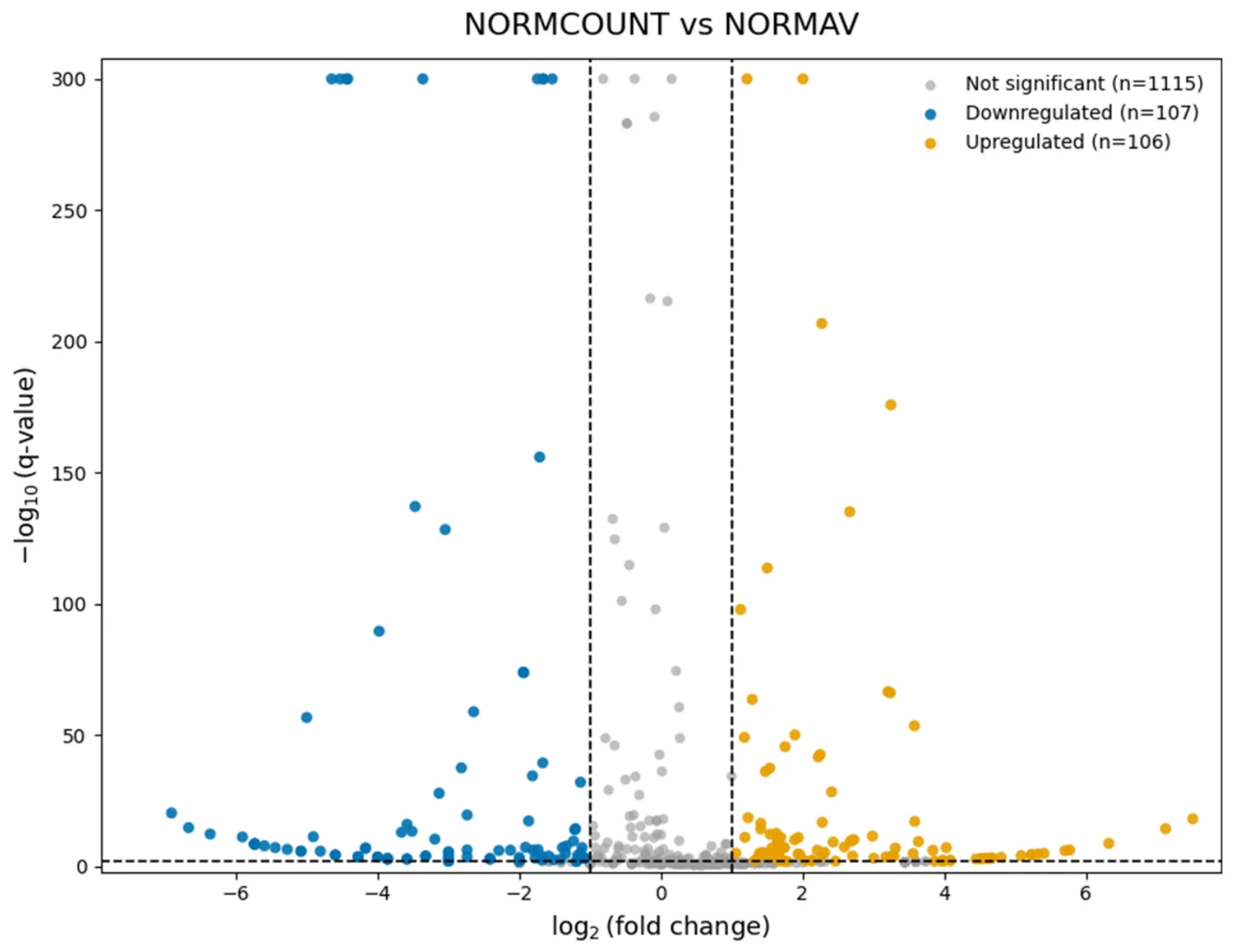
Volcano plot of differentially expressed miRNAs between the NORMCOUNT and NORMAV groups. The x-axis represents log_2_ fold change (NORMCOUNT vs. NORMAV), and the y-axis represents −log_10_(*q-value*). MiRNAs with *q* < 0.01 and |log_2_FC| > 1 were considered significantly differentially expressed. Upregulated and downregulated miRNAs are shown separately, whereas non-significant miRNAs are shown in grey. Dashed lines indicate the predefined significance thresholds. For visualization purposes, *q-values* equal to zero were displayed using an upper cap of −log_10_(*q*) = 300; this graphical transformation did not affect statistical significance or differential-expression classification.

Differential expression analysis was initially performed between normozoospermic samples characterized by markedly elevated sperm concentration (NORMCOUNT) and those representing the investigator-defined intermediate normozoospermic phenotype (NORMAV). This analysis identified a total of 213 significantly differentially expressed (DE) miRNAs, comprising 106 upregulated and 107 downregulated miRNAs in the NORMCOUNT group (Figure 3, Table S2).

Among the most strongly upregulated miRNAs, hsa-miR-891b exhibited the highest positive fold change, followed by hsa-miR-34c-3p and the novel candidate miRNA novel_46. Conversely, the strongest downregulation was observed for hsa-miR-142-3p, followed by novel_75 and hsa-miR-145-5p (Table 4). Overall, these findings revealed pool-level differences in sperm miRNA abundance between normozoospermic men characterized by high sperm concentration and those representing the investigator-defined intermediate normozoospermic phenotype.

**Figure 4:**
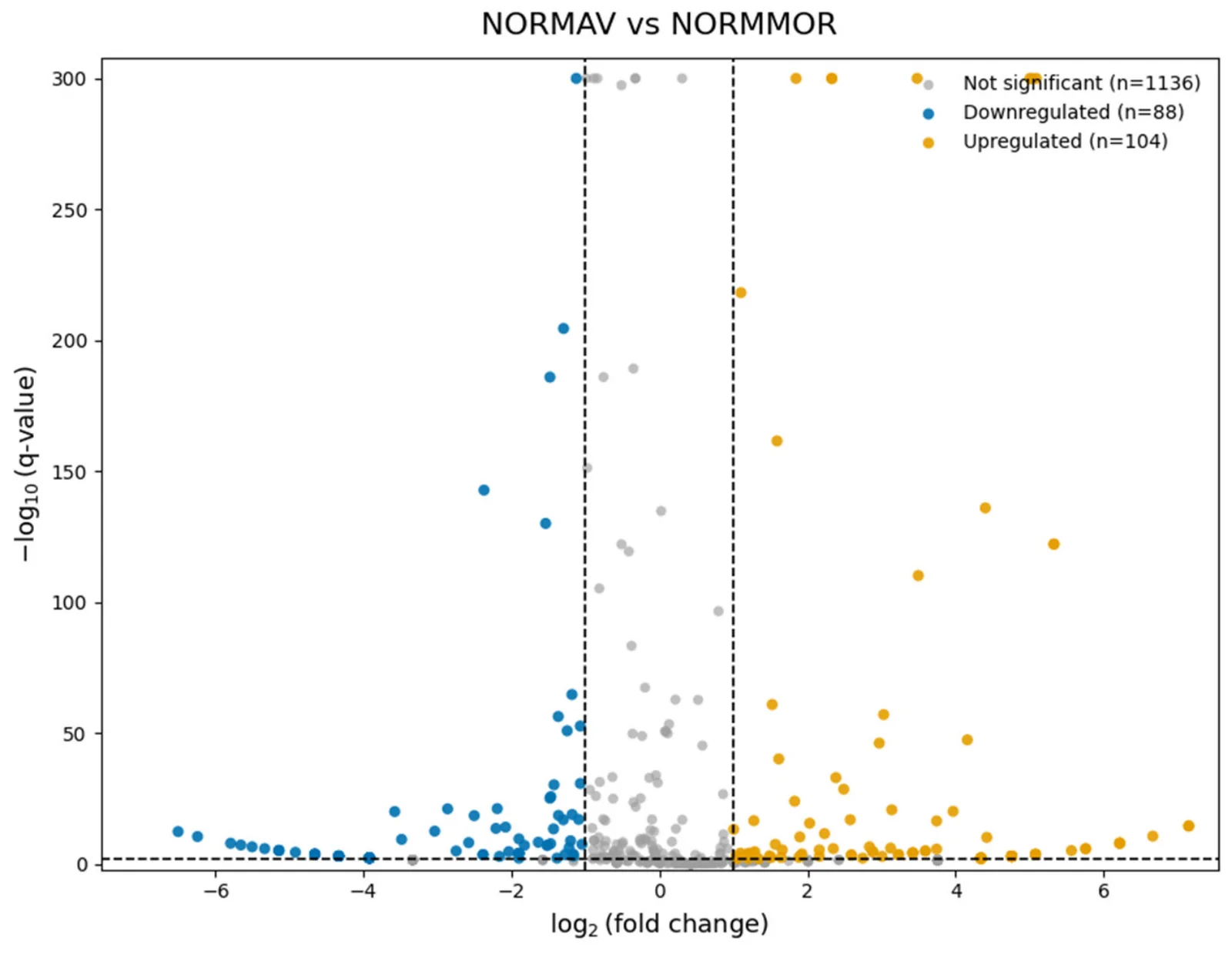
Volcano plot of differentially expressed miRNAs between the NORMAV and NORMMOR groups. The x-axis represents the log_2_ fold change (NORMAV vs. NORMMOR), while the y-axis represents the −log_10_(*q-value*). Differentially expressed miRNAs were defined using a *q-value* < 0.01 and |log_2_FC| > 1. A total of 104 miRNAs were upregulated and 88 were downregulated in NORMAV relative to NORMMOR. Upregulated and downregulated miRNAs are shown in orange and blue, respectively, whereas non-significant miRNAs are shown in grey. Dashed vertical and horizontal lines indicate the log_2_FC (±1) and *q-value* (0.01) significance thresholds, respectively. For visualization purposes, −log_10_(*q-value*) values exceeding 300, including cases where the reported *q-value* was numerically zero, were capped at 300.

The second differential expression analysis was conducted between normozoospermic samples representing the investigator-defined intermediate normozoospermic phenotype (NORMAV) and those characterized by a high proportion of morphologically normal spermatozoa (NORMMOR). A total of 192 miRNAs were identified as significantly differentially expressed, with 104 upregulated and 88 downregulated in the NORMAV group (Figure 4, Table S2).

Among the most strongly upregulated miRNAs, hsa-miR-1-3p and the novel candidate miRNA novel_75 exhibited the highest positive fold changes, followed by hsa-miR-889-3p. Conversely, hsa-miR-891b showed the strongest downregulation, followed by hsa-miR-34c-3p and hsa-miR-664a-3p (Table 5). Notably, hsa-miR-891b and hsa-miR-34c-3p were among the most differentially expressed miRNAs in both the NORMCOUNT–NORMAV and NORMAV–NORMMOR comparisons; considered together, both were more abundant in NORMCOUNT and in NORMMOR than in NORMAV. The novel candidate novel_75 was also recurrent, being less abundant in NORMCOUNT than in NORMAV and more abundant in NORMAV than in NORMMOR. These three-group abundance patterns indicate recurrent differential expression of these miRNAs across the normozoospermic phenotypes examined.

**Figure 5:**
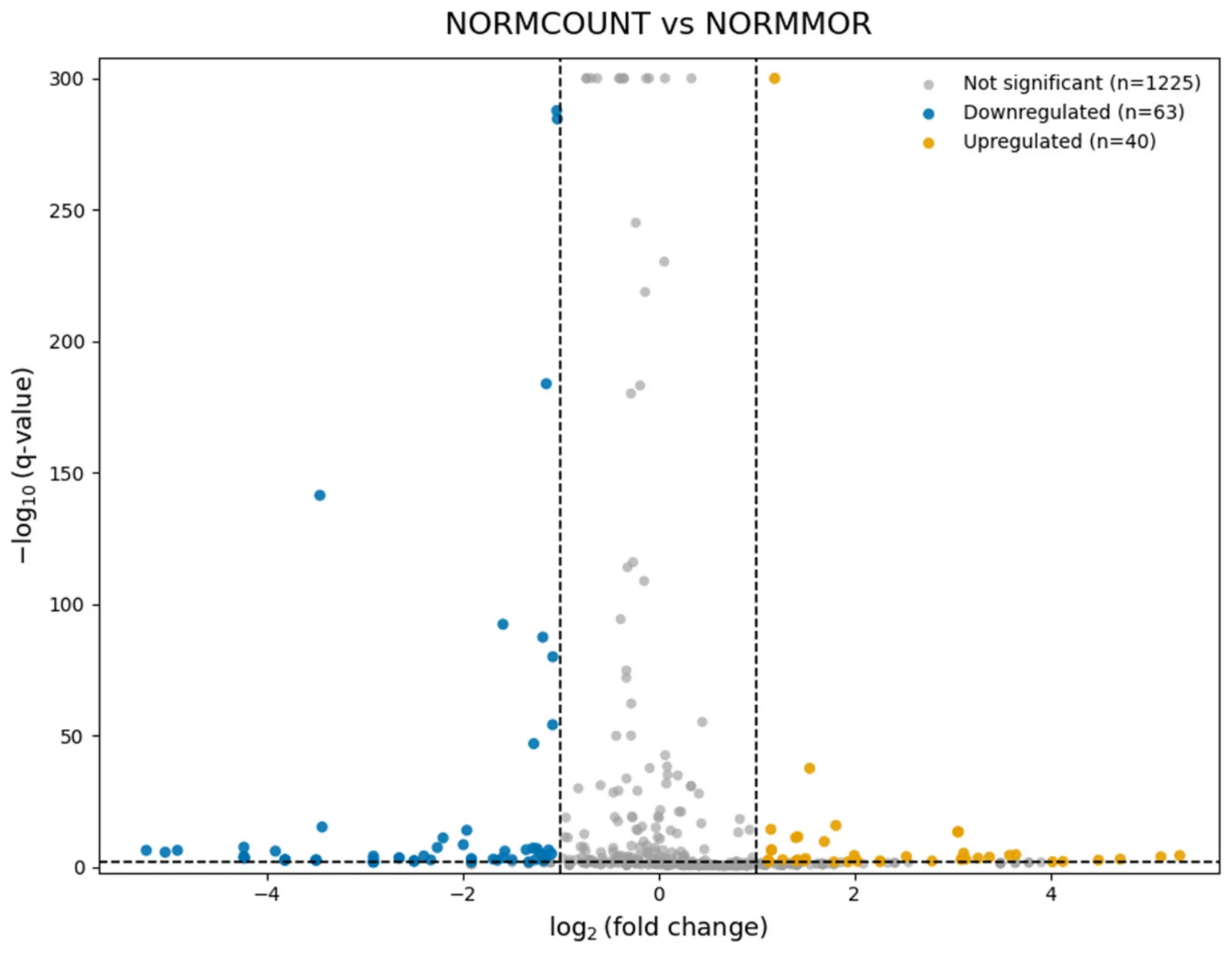
Volcano plot of differentially expressed miRNAs between the NORMCOUNT and NORMMOR groups. The x-axis represents the log_2_ fold change (log_2_FC) (NORMCOUNT vs. NORMMOR), while the y-axis represents the −log_10_-transformed *q-value*. Differentially expressed miRNAs were identified using thresholds of |log_2_FC| > 1 and *q-value* < 0.01. A total of 103 miRNAs were differentially expressed, including 40 upregulated and 63 downregulated miRNAs in NORMCOUNT relative to NORMMOR. miRNAs not meeting both significance criteria are shown as non-significant. For visualization purposes, *q-values* equal to zero were assigned a minimum plotting value, resulting in an upper cap for −log_10_(*q-value*); this adjustment affected only graphical representation and not statistical significance or classification of differentially expressed miRNAs.

The third differential expression analysis compared normozoospermic samples characterized by high sperm concentration (NORMCOUNT) with those exhibiting a high proportion of morphologically normal spermatozoa (NORMMOR). A total of 103 miRNAs were identified as significantly differentially expressed, of which 40 were upregulated and 63 were downregulated in the NORMCOUNT group (Figure 5, Table S2).

Among the most strongly upregulated miRNAs, hsa-miR-1-3p exhibited the highest positive fold change, followed by hsa-miR-106a-3p and hsa-miR-708-3p. Conversely, the strongest downregulation was observed for hsa-miR-145-5p, followed by hsa-miR-23b-5p and hsa-miR-411-5p (Table 6). Overall, fewer differentially expressed miRNAs were identified between NORMCOUNT and NORMMOR than in comparisons involving the NORMAV group.

**Figure 6:**
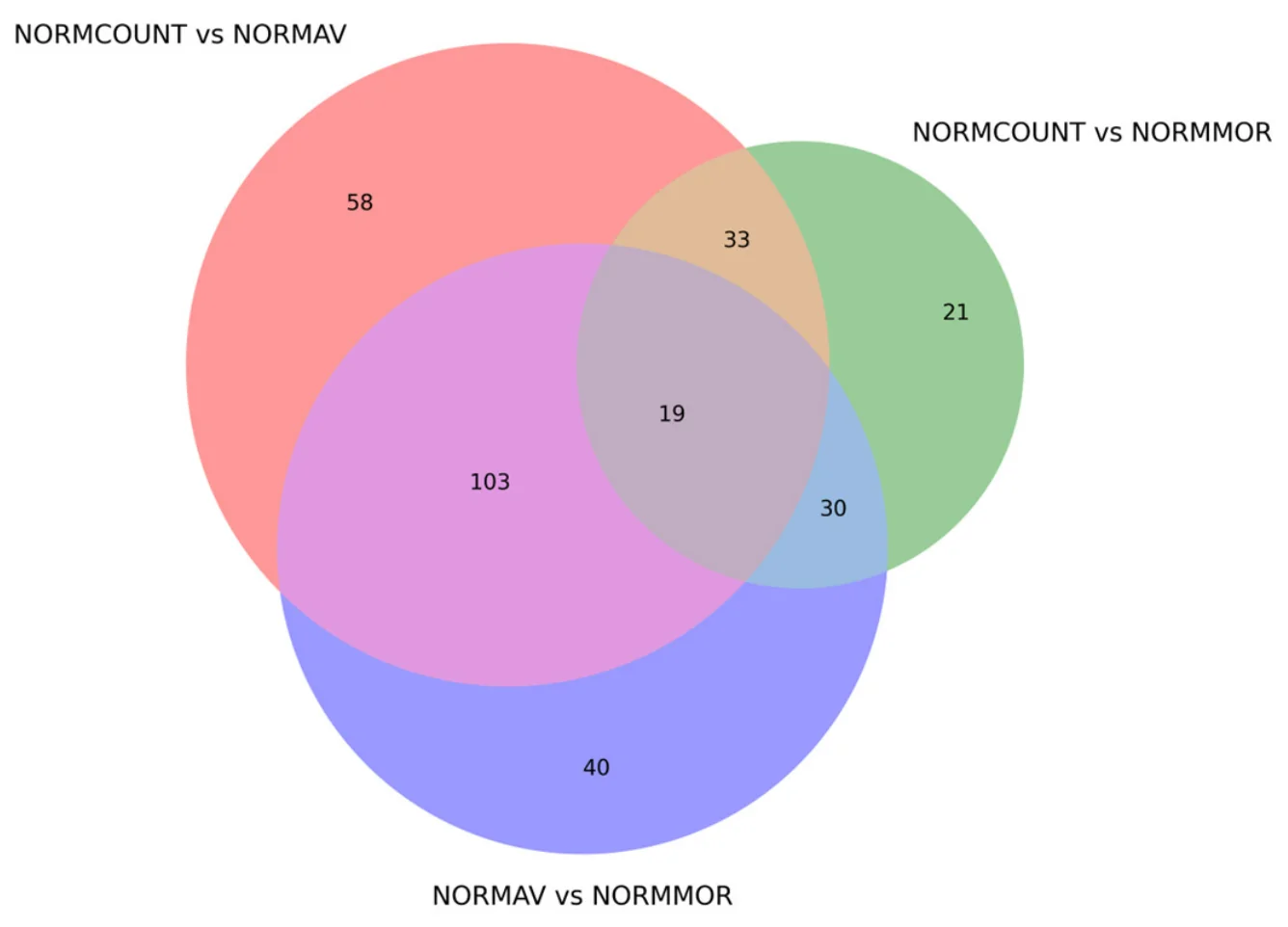
Overlap of differentially expressed miRNAs among the three pairwise comparisons. Venn diagram showing shared and comparison-specific differentially expressed miRNAs identified in NORMCOUNT vs. NORMAV, NORMCOUNT vs. NORMMOR, and NORMAV vs. NORMMOR.

Overlap analysis of the differentially expressed miRNAs revealed both comparison-specific and recurrent expression changes across the three investigator-defined normozoospermic groups (Figure 6). A core set of 19 miRNAs met the differential-expression criteria in all three pairwise comparisons, suggesting their association with variation among the investigated normozoospermic groups (Table S3). Furthermore, 103 miRNAs were exclusively shared between the NORMCOUNT–NORMAV and NORMAV–NORMMOR comparisons, while 33 and 30 miRNAs were shared between NORMCOUNT–NORMAV/NORMCOUNT–NORMMOR and NORMCOUNT–NORMMOR/NORMAV–NORMMOR, respectively. Comparison-specific expression changes were also observed, with 58, 21, and 40 miRNAs uniquely identified in the NORMCOUNT–NORMAV, NORMCOUNT–NORMMOR, and NORMAV–NORMMOR comparisons, respectively.

### 3.4. Target Gene Identification and Functional Enrichment Analysis of Differentially Expressed miRNAs

To characterize the biological annotations associated with the observed miRNA differences, an in silico target-gene analysis was performed for each pairwise comparison. Gene Ontology [41,42] and KEGG [43] pathway enrichment analyses were subsequently performed to identify the biological processes, cellular components, and molecular functions associated with these database-curated miRNA target genes.

**Figure 7:**
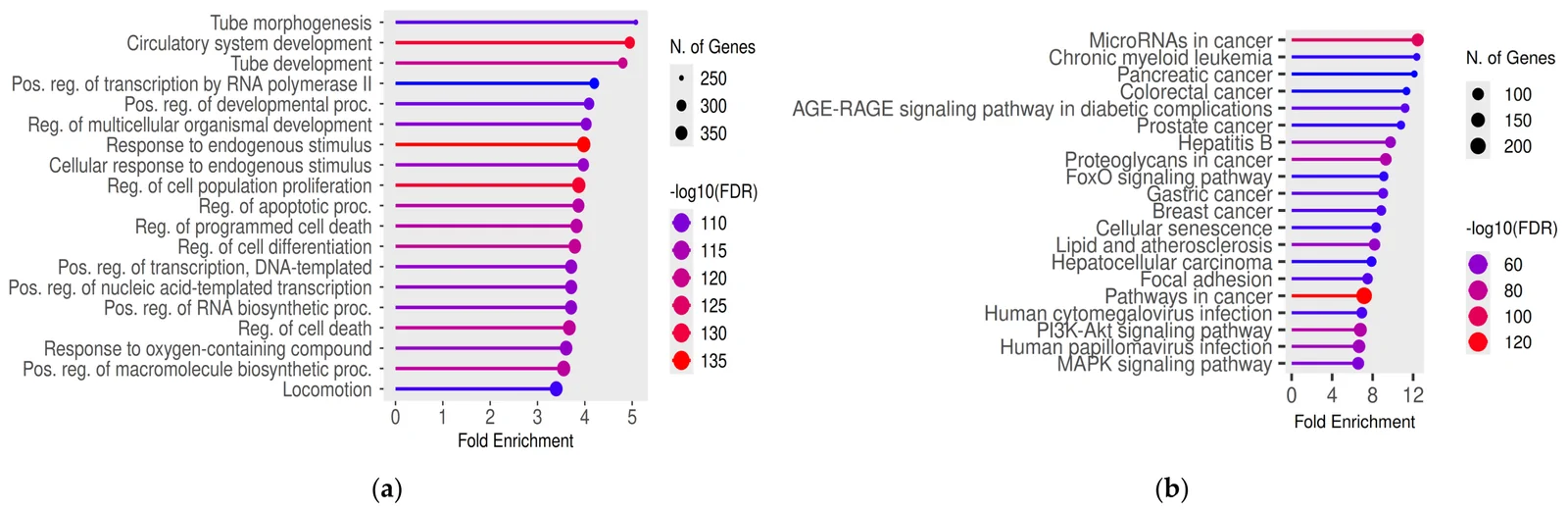
Significant (**a**) GO Biological Process and (**b**) KEGG pathway terms associated with the overlap gene targets of DE miRNAs for the NORMCOUNT vs. NORMAV comparison. The size and color of the dots represent the number of genes and the range of statistical significance, respectively. The red color indicates higher −log10(FDR) values, followed by pink, purple and blue colors. The y-axis represents the GO and KEGG terms, and the x-axis represents fold enrichment. The *p-values* were corrected for multiple tests using the false discovery rate (FDR).

At first, for the NORMCOUNT vs. NORMAV comparison, analysis of experimentally validated miRNA–target interactions identified 1342 unique overlapping target genes, with an average of 16.57 targets per miRNA (Table S4). GO enrichment revealed significant representation of terms related to developmental and cellular regulatory processes. Within the Biological Process category, prominently enriched terms included tube morphogenesis, tube development, and circulatory system development. Enriched terms also encompassed the regulation of transcription and RNA biosynthesis, cell population proliferation and differentiation, apoptosis and programmed cell death, as well as cellular responses to endogenous stimuli and oxygen-containing compounds (Figure 7a). GO Cellular Component (CC) analysis highlighted transcription regulator complexes, membrane rafts and microdomains, cell–substrate and cell–cell junctions, focal adhesions, as well as chromatin-, chromosome-, and nucleoplasm-associated compartments (Figure S1). GO Molecular Function (MF) analysis was dominated by functions related to transcriptional regulation and kinase-associated activity, including DNA-binding transcription factor binding, transcription factor binding, DNA-binding transcription activator activity, protein kinase binding, and kinase binding/activity (Figure S2). KEGG enrichment further identified several signaling-related pathways, notably FoxO, MAPK, and PI3K-Akt signaling, along with focal adhesion and cellular senescence (Figure 7b).

**Figure 8:**
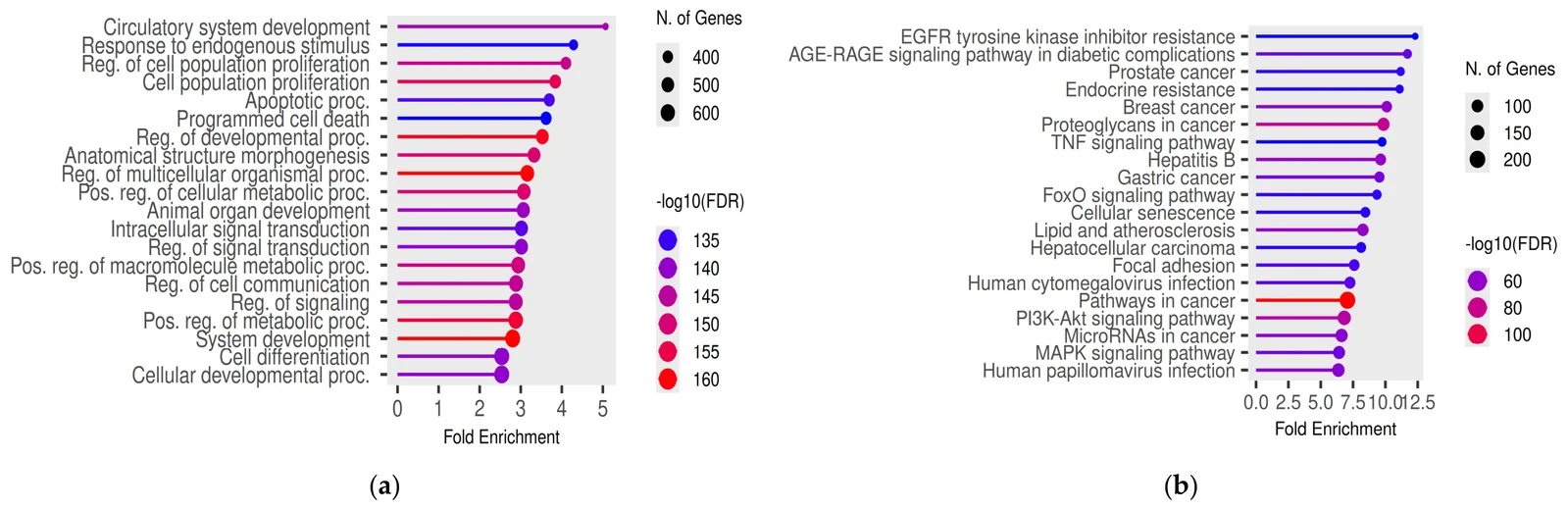
Significant (**a**) GO Biological Process and (**b**) KEGG pathway terms associated with the overlap gene targets of DE miRNAs for the NORMAV vs. NORMMOR comparison. The size and color of the dots represent the number of genes and the range of statistical significance, respectively. The red color indicates higher −log10(FDR) values, followed by pink, purple and blue colors. The y-axis represents the GO and KEGG terms, and the x-axis represents fold enrichment. The *p*-values were corrected for multiple tests using the false discovery rate (FDR).

For the NORMAV vs. NORMMOR comparison, 1254 unique overlapping experimentally validated target genes were identified and retained for functional enrichment analysis, with an average of 16.56 targets per miRNA (Table S5). GO Biological Process enrichment analysis highlighted processes related to developmental regulation and morphogenesis, cell proliferation and programmed cell death, as well as intracellular signal transduction, cell communication, and metabolic regulation (Figure 8a). Cellular Component analysis showed enrichment in transcription regulator complexes, membrane rafts and microdomains, focal and cell–substrate adhesions, cell–cell and anchoring junctions, and chromatin-associated compartments (Figure S3). Molecular Function terms were predominantly associated with transcription factor and DNA binding, protein kinase binding and activity, and other transcriptional regulatory functions (Figure S4). KEGG pathway analysis further indicated enrichment of major signaling and cellular regulatory pathways, including PI3K–Akt, MAPK, FoxO, and TNF signaling, together with focal adhesion and cellular senescence pathways. Several cancer-related pathways were also significantly enriched, potentially reflecting the extensive involvement of the identified target genes in shared regulatory mechanisms controlling cell proliferation, survival, differentiation, and signal transduction (Figure 8b).

**Figure 9:**
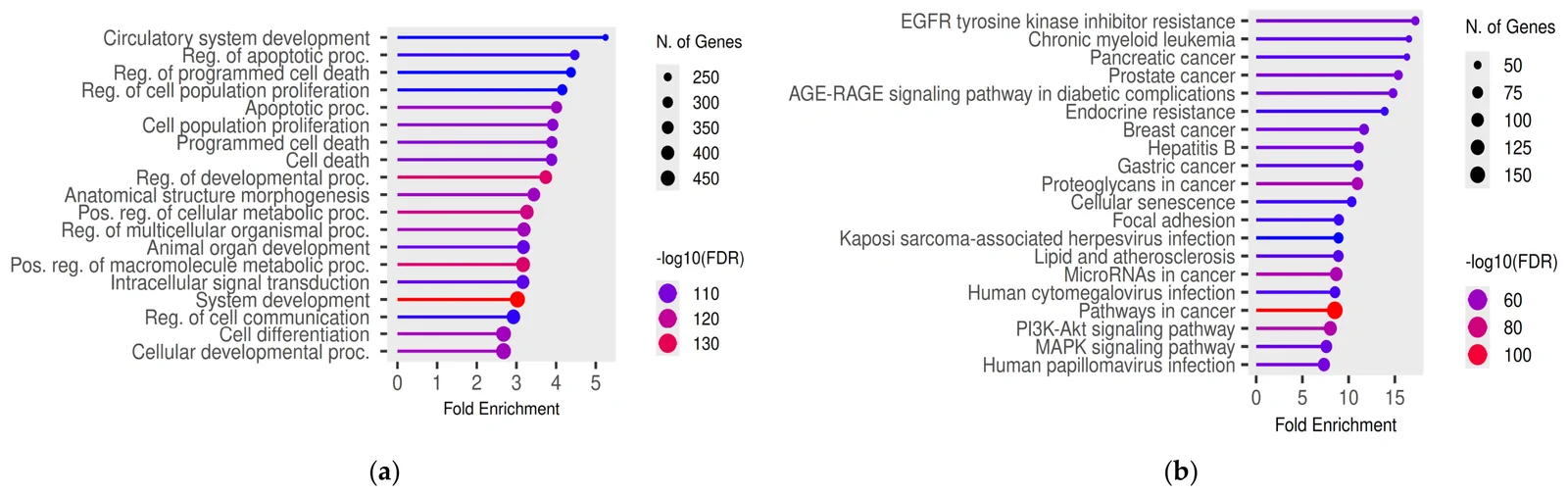
Significant (**a**) GO Biological Process and (**b**) KEGG pathway terms associated with the overlap gene targets of DE miRNAs for the NORMCOUNT vs. NORMMOR comparison. The size and color of the dots represent the number of genes and the range of statistical significance, respectively. The red color indicates higher −log10(FDR) values, followed by pink, purple and blue colors. The y-axis represents the GO and KEGG terms, and the x-axis represents fold enrichment. The *p-values* were corrected for multiple tests using the false discovery rate (FDR).

For the NORMCOUNT vs. NORMMOR comparison, 898 unique experimentally validated target genes were retained for functional enrichment analysis, corresponding to an average of 21.13 target genes per miRNA (Table S6). GO Biological Process (BP) enrichment was predominantly associated with the regulation of cell proliferation and cell death, including apoptotic and programmed cell death processes, together with developmental and differentiation-related processes. Enrichment was also observed for intracellular signal transduction, regulation of cell communication, and cellular and macromolecule metabolic processes (Figure 9a). GO Cellular Component (CC) analysis highlighted transcription regulator complexes, membrane rafts and microdomains, focal and cell–substrate adhesions, receptor complexes, chromatin, and cell junction-associated compartments (Figure S5). Molecular Function (MF) terms were primarily related to transcription factor and transcription activator binding, protein kinase/kinase binding and activity, enzyme binding, and sequence-specific DNA binding (Figure S6). KEGG analysis revealed enrichment of major signaling pathways, including PI3K–Akt and MAPK signaling, together with focal adhesion and cellular senescence, while several cancer-related pathways were also represented (Figure 9b).

To identify genes recurrently listed as validated targets across the three pairwise comparisons, strongly validated miRNA–target interactions were further examined. After collapsing duplicate experimental records for individual miRNA–target pairs, 568 target genes were shared across all three comparisons (Table S7). Several genes were targeted by multiple differentially expressed miRNAs, indicating substantial convergence of miRNA-mediated regulation. *VEGFA* represented the most recurrent target, being targeted by 21, 21, and 11 DE miRNAs in the NORMCOUNT vs. NORMAV, NORMAV vs. NORMMOR, and NORMCOUNT vs. NORMMOR comparisons, respectively. Other recurrently targeted genes included *IGF1R* (15, 17, and 11 miRNAs), *BCL2* (17, 15, and 10), *PTEN* (18, 15, and 4), *CDK6* (13, 10, and 10), *MYC* (11, 12, and 8), *MET* (11, 10, and 8), and *CCND1* (9, 13, and 7). Collectively, these database-derived observations identify a set of genes recurrently listed as validated targets of distinct DE miRNAs across normozoospermic subgroups.

## 4. Discussion

The present pilot pooled-sequencing study identified pool-level differences in sperm miRNA abundance among three investigator-defined groups of normozoospermic men defined by selected combinations of sperm concentration and normal morphology. These observations suggest potential molecular variation among the investigated concentration- and morphology-based profiles but do not establish an independent association of miRNA abundance with either sperm concentration or morphology. Comparative small RNA profiling of normozoospermic men representing an intermediate phenotype, a high-sperm-concentration phenotype, or a high-normal-morphology phenotype revealed distinct miRNA expression patterns among the three subgroups. The corresponding database-derived lists of validated target genes yielded recurrent GO and KEGG annotations related to transcriptional regulation, cell proliferation and death, development, and intracellular signaling. Furthermore, a set of target genes was recurrently identified across all comparisons, raising the hypothesis that different miRNA profiles may converge on common regulatory nodes. These exploratory observations support further investigation of molecular variation within normozoospermia at the individual-sample level.

Previous studies have challenged the concept of normozoospermia as a molecularly homogeneous condition, as evidenced by Salas-Huetos et al. (2016) [9], Denomme et al. (2017) [27] and Santiago et al. (2023) [29]. Although the available evidence remains limited, these observations suggest that normozoospermia may represent a heterogeneous molecular phenotype. Furthermore, numerous studies report altered sperm miRNA profiles across oligozoospermic, asthenozoospermic, and teratozoospermic phenotypes, with specific miRNAs associated with conventional semen characteristics, including sperm concentration, motility, and morphology [18,20]. The present study extends these observations by identifying pool-level differences in sperm miRNA abundance among selected investigator-defined groups within normozoospermia, defined according to sperm concentration and normal morphology. These pool-level observations suggest that miRNA-associated molecular variation may not be restricted to comparisons between normal and pathological semen profiles but may also be detectable among concentration- and morphology-based groups within the conventionally defined normal range. However, because sperm concentration and morphology were considered jointly in defining these groups, the observed pool-level miRNA differences cannot be attributed independently to either semen characteristic. Rather, the findings generate hypotheses regarding molecular variation among the predefined concentration- and morphology-based profiles that require confirmation at the individual-sample level.

Among the differentially expressed miRNAs, several recurrent candidates emerged across pairwise comparisons, highlighting specific miRNAs as candidates for subsequent individual-level validation. Particularly notable was miR-34c-3p, which was more abundant in both NORMCOUNT and NORMMOR than in NORMAV; the two contrasts therefore showed a concordant direction relative to NORMAV rather than opposing patterns. Members of the miR-34 family have been extensively implicated in male germ-cell development, spermatogenesis, sperm function, and early embryonic development, while altered sperm miR-34c abundance has been associated with impaired semen characteristics and unexplained male infertility [45]. An early comprehensive analysis of sperm miRNA profiles conducted by Abu-Halima et al. (2013) [26] revealed extensive miRNA dysregulation in asthenozoospermic and oligoasthenozoospermic men when compared to normozoospermic controls. Specifically, miR-34c-5p exhibited pronounced differential expression in oligoasthenozoospermic men, an observation subsequently validated by qRT-PCR, thereby supporting an association between the miR-34c locus and impaired semen parameters [26]. Interestingly, the recurrent differential expression of miR-34c-3p across our comparisons suggests that miR-34c-associated regulation may extend beyond the distinction between normal and impaired semen phenotypes, potentially encompassing quantitative variations in sperm characteristics even within the normozoospermic range. Similarly, miR-891b, another recurrent candidate, showed the same pattern, being more abundant in both NORMCOUNT and NORMMOR relative to NORMAV. Notably, miR-891b belongs to a primate-specific, X-linked epididymal miRNA cluster previously implicated in unexplained asthenozoospermia in a study of the cell-free seminal-plasma compartment rather than spermatozoa, which may suggest an association with post-testicular sperm maturation and function [46]. miR-145-5p was also consistently identified, being less abundant in NORMCOUNT than in both NORMAV and NORMMOR, whereas miR-1-3p was more abundant in NORMAV and NORMCOUNT than in NORMMOR; both should be regarded as exploratory candidates pending individual-level validation. Beyond these recurrent top-ranking miRNAs, several additional DE miRNAs identified in the present dataset have previously been associated with sperm quality or male infertility. Specifically, miR-9-3p and miR-122-5p have consistently demonstrated differential expression across various male infertility phenotypes and have been independently associated with sperm concentration and motility, thus reinforcing their potential relevance to sperm quality [47]. Members of the miR-23 family have likewise been implicated in the regulation of male germ-cell and sperm-related processes and have also been correlated with basic semen parameters [48]. Collectively, the recurrence of these previously reported sperm-associated miRNAs, alongside the differential abundance patterns observed across the pooled comparisons, is consistent with the biological plausibility of the observed differences and suggests the existence of molecular variation across the investigated phenotypic groups within normozoospermia.

Functional analysis of the strongly validated miRNA targets also revealed recurrent enrichment annotations across the three pairwise comparisons. Across the pool-level miRNA comparisons involving NORMAV, NORMCOUNT, and NORMMOR, GO enrichment repeatedly highlighted processes related to cell proliferation, apoptosis and programmed cell death, development and differentiation, intracellular signaling, and transcriptional regulation. Similarly, KEGG analysis consistently identified major signaling networks, particularly PI3K–Akt and MAPK signaling, together with FoxO signaling, focal adhesion, and cellular senescence. These recurrent annotations generate hypotheses but do not demonstrate altered pathway activity in mature sperm or during spermatogenesis, particularly as up- and downregulated miRNAs were analyzed jointly; they are nevertheless of interest because miRNA-mediated post-transcriptional regulation is critically involved in spermatogonial stem-cell self-renewal and differentiation, meiotic progression, spermatid differentiation, and the proliferation and maturation of Sertoli cells [49,50].

Among the recurrently enriched pathways, PI3K–Akt signaling is particularly relevant to male reproductive biology. The PI3K/AKT/mTOR axis participates at multiple stages of spermatogenesis, contributing to the balance between spermatogonial stem-cell maintenance and differentiation, germ-cell survival, Sertoli-cell proliferation and metabolism, and testicular endocrine function [51,52]. The relationship with FoxO signaling observed in our enrichment results is also noteworthy, as FOXO1 activity is tightly regulated by PI3K/AKT and PTEN and contributes to spermatogonial stem-cell homeostasis and the initiation of spermatogenesis [53,54]. MAPK signaling was another recurrently enriched annotation. Beyond its involvement in Sertoli-cell proliferation and germ-cell development, MAPK signaling contributes to the dynamic regulation of testicular cell–cell junctions and has been implicated in later sperm functions, including epididymal maturation, capacitation, and the acrosome reaction [55,56]. Thus, the repeated enrichment of these pathways across the present comparisons suggests that the observed miRNA differences may relate to regulatory networks with established roles throughout male germ-cell development and sperm function. Importantly, it should be noted that the functional enrichment analyses performed here are hypothesis-generating and should not be interpreted as evidence that the identified pathways are functionally altered in sperm or during fertilization and embryonic development. The GO and KEGG results reflect annotations associated with database-curated validated targets of the differentially abundant miRNAs rather than direct measurements of target-gene expression or pathway activity in the samples analyzed. Functional studies will therefore be required to determine whether the candidate miRNAs identified here exert regulatory effects relevant to sperm function or post-fertilization processes.

At the target level, 568 genes were listed in all three comparisons, with several genes independently targeted by multiple DE miRNAs. *VEGFA* emerged as the most recurrent target, followed by genes including *IGF1R*, *BCL2*, *PTEN*, *CDK6*, *MYC*, *MET*, and *CCND1*. The prominence of *VEGFA* is particularly noteworthy within the context of spermatogenesis. Experimental disruption of testicular *Vegfa* in mice has been associated with subfertility, reduced sperm counts, and alterations in the undifferentiated spermatogonial population, thereby supporting a critical role for VEGFA signaling in spermatogonial homeostasis [57]. More recently, Özbek et al. (2025) [58] demonstrated a highly dynamic and cell-specific expression pattern of VEGF and its receptors throughout postnatal testicular and epididymal development. VEGF expression was detected in Leydig cells across all developmental stages and, following puberty, in Sertoli cells and specific germ-cell populations, including spermatogonia and elongated spermatids. Concomitantly, VEGF receptors also exhibited developmentally regulated expression in the testis and epididymis [58]. These observations further support a role for VEGF signaling in spermatogonial proliferation and differentiation, testicular development, and epididymal physiology. Importantly, VEGF receptor activation engages downstream signaling cascades including PI3K/AKT/mTOR and RAS/RAF/MEK/ERK [58], which may offer a hypothetical link between the recurrent listing of *VEGFA* observed in the present study and the consistent enrichment of PI3K–Akt and MAPK signaling pathways across our comparisons.

Similarly, insulin/IGF signaling plays a crucial role in testicular development and spermatogenesis. Experimental models have demonstrated that the disruption of insulin and IGF1 receptors in Sertoli cells leads to a significant reduction in Sertoli-cell proliferation, adult testis size, and daily sperm production [59,60]. Pertinently, regarding the NORMCOUNT phenotype examined in this study, Cannarella et al. (2022) [61] observed a positive correlation between the serum IGF1/FSH ratio and sperm concentration, total sperm count, and testicular volume in men undergoing semen analysis. Furthermore, individuals with oligozoospermia exhibited a significantly lower IGF1/FSH ratio compared to those with a normal total sperm count. Notably, IGF1 levels alone were not significantly associated with sperm concentration or total sperm count, suggesting that this relationship may reflect the broader interplay between IGF1 and gonadotropic signaling rather than circulating IGF1 levels per se [61]. Within this framework, the recurrent targeting of *IGF1R* by DE miRNAs across the present comparisons, particularly those involving differences in sperm concentration, is consistent with, but does not establish, the involvement of IGF-related regulatory networks in the molecular heterogeneity observed within normozoospermia.

*PTEN* was also recurrently listed as a target, in keeping with the PI3K–Akt annotations consistently observed across our enrichment analyses. Specifically, PTEN functions as a major negative regulator of PI3K/AKT signaling. Experimental studies have demonstrated that appropriate PTEN activity is indispensable for the long-term maintenance of spermatogonial stem cells and the precise regulation of spermatogenesis [62]. Corroborating this, PI3K–AKT activation plays a pivotal role in spermatogonial stem-cell self-renewal, underscoring the importance of tightly regulated signaling within this pathway [51]. PTEN has also been implicated in the regulation of IGF-dependent Sertoli-cell proliferation [63], thereby providing a further conceptual connection between recurrently listed targets such as *PTEN* and *IGF1R* in the present dataset.

Taken together, these findings indicate pool-level miRNA differences among distinct normozoospermic phenotypes and point to candidate regulatory networks that may contribute to this molecular heterogeneity. Nevertheless, the exploratory nature of the present study should be considered when interpreting these observations, and several aspects warrant further investigation. A major limitation of this pilot pooled-sequencing study is that differential-expression inference was based on only six pooled libraries (two per group), and the identified miRNA differences were not subsequently validated by RT-qPCR in the individual samples. Consequently, the observed pool-level fold changes cannot establish whether these differences were consistently present across participants or were influenced by one or a few individuals within a pool. The findings therefore apply to pooled libraries and cannot estimate individual-level variability or adjust for participant-level confounders such as age, abstinence, body mass index, or smoking. Although pool composition was designed to generate broadly comparable biological replicates and all six libraries were prepared in the same batch, sequenced in the same lane, and processed with randomized library order, the use of only two pooled libraries per group means that residual pool-level biological or technical effects cannot be completely excluded. Future studies employing larger cohorts and individual-level sequencing will be necessary to confirm the observed expression patterns and determine their robustness across independent populations. Second, the identified DE miRNAs were not independently validated using an orthogonal method such as RT-qPCR. Regarding the functional analyses, they should also be interpreted within the context of the study design. Although only strongly experimentally validated miRNA–target interactions were considered, the expression of the corresponding target genes was not directly measured in the present samples. Consequently, the enrichment of pathways such as PI3K–Akt, MAPK, and FoxO and the recurrent targeting of genes including *VEGFA*, *IGF1R*, and *PTEN* indicate potential regulatory convergence rather than demonstrated alterations in pathway activity or target-gene expression. Importantly, these enrichment results do not demonstrate functional effects of the identified miRNAs in sperm, fertilization, or embryonic development. Rather, they represent hypothesis-generating annotations based on database-curated target interactions, and dedicated functional experiments will be required to establish whether the candidate miRNAs exert such effects in the reproductive context. Moreover, up- and downregulated miRNAs were analyzed jointly, and database-curated interactions are not specific to the sperm or testicular context, so neither the direction nor the tissue relevance of any putative effect can be inferred. Future studies integrating sperm miRNA sequencing with transcriptomic and proteomic profiling could determine whether the observed miRNA differences are accompanied by corresponding changes in their target genes and downstream signaling networks. Another limitation relates to the predefined grouping strategy. Because sperm concentration and normal morphology were considered jointly in defining the three groups, the present design cannot determine whether the observed pool-level miRNA differences are independently associated with sperm concentration, morphology, or their combination. The groups should therefore be interpreted as selected concentration- and morphology-based profiles rather than as representations of holistic semen phenotypes. Future individual-level studies should evaluate sperm concentration and morphology separately, including their analysis as continuous variables, to determine whether specific miRNA differences are independently associated with either semen characteristic. An additional limitation concerns the scope of the phenotypic stratification. The present study focused specifically on investigator-defined groups based on sperm concentration and normal morphology and was not designed to capture the full spectrum of semen phenotypes defined by conventional semen parameters, including variation in semen volume and motility. Although these parameters were assessed and remained within the WHO reference criteria in all participants, they were not used as independent axes of stratification. Therefore, the observed miRNA differences should be interpreted as molecular variation among the specific concentration- and morphology-based groups investigated here, rather than as representative of molecular heterogeneity across the complete spectrum of semen phenotypes. Importantly, the biological and clinical significance of the observed pool-level miRNA differences remains to be established. The present study did not assess fertilization, embryo development, pregnancy, live birth, or other measures of reproductive potential and therefore cannot determine whether the identified candidate miRNAs have predictive value beyond conventional semen analysis. Future studies should evaluate these candidates at the individual level in larger, independent cohorts and relate their abundance to direct measures of sperm function and prospectively collected reproductive outcomes. Only after such validation could the potential complementary value of sperm miRNA profiling for reproductive assessment or infertility diagnostics be determined.

Beyond validation of the present findings, several directions could further elucidate the biological and clinical significance of molecular heterogeneity within normozoospermia. Replication in larger, independent cohorts with individual-level sequencing will be essential to determine whether the identified miRNA signatures consistently differentiate normozoospermic men across phenotypes characterized by average, high-concentration, and high-morphology sperm. Rather than exclusively relying on predefined categories, future studies could also examine sperm concentration and morphology as continuous variables to ascertain whether miRNA abundance follows quantitative gradients across the normal semen range. Future studies incorporating larger cohorts and independent stratification across additional semen parameters will be required to determine whether comparable miRNA patterns extend to other phenotypic dimensions within normozoospermia. Longitudinal sampling would further help establish whether these molecular profiles represent stable individual characteristics or fluctuate in response to temporal, lifestyle, or environmental factors. Functional studies focusing on recurrent candidate miRNAs and their validated targets, including *VEGFA*, *IGF1R*, and *PTEN*, could determine whether the database-derived regulatory relationships highlighted by the present analysis have functional relevance in sperm or post-fertilization processes. Finally, integrating sperm miRNA profiles with direct measures of sperm function, including DNA fragmentation, oxidative stress, capacitation, and fertilization competence, and linking these molecular profiles to clinically relevant reproductive outcomes, such as fertilization, embryo development, pregnancy and live birth rates, will be particularly important. Such studies will be necessary to determine whether the candidate miRNAs show reproducible associations with reproductive outcomes and whether, after appropriate validation, sperm miRNA profiling could provide complementary information beyond conventional semen analysis. The present data do not support its use for prediction of reproductive potential or modification of current infertility diagnostic criteria.

## 5. Conclusions

This pilot pooled-sequencing study identified differences in sperm miRNA abundance among pooled samples representing investigator-defined normozoospermic groups based on sperm concentration and normal morphology. The observed pool-level differences highlight several candidate miRNAs and associated regulatory pathways that warrant further investigation but should not be interpreted as established molecular signatures at the individual level. Given the pooled design and the absence of individual-level molecular validation, the present findings are exploratory and hypothesis-generating. Individual-sample sequencing and orthogonal validation in larger, independent cohorts will be required to determine whether the candidate miRNA differences identified here are consistently observed across individuals and whether they are associated with specific semen characteristics. Integration with sperm functional measures and reproductive outcomes will ultimately be necessary to assess their potential biological and clinical relevance.

## Figures and Tables

**Table 1:** Demographic, lifestyle, and semen characteristics of participants across the three normozoospermic phenotypic groups. Continuous variables are presented as mean ± standard deviation (SD). For semen parameters, participant-level values represent the mean of the two independent semen assessments. Between-group comparisons were performed using Welch’s one-way ANOVA for continuous variables, except for abstinence duration, which was analyzed using the Kruskal–Wallis test. Smoking status and alcohol consumption were compared using the chi-square test. A *p-value* < 0.05 was considered statistically significant.

Characteristic	NORMAV (*n* = 10)	NORMCOUNT (*n* = 10)	NORMMOR (*n* = 10)	*p-Value*
Age (years)	37.80 ± 4.10	38.30 ± 6.91	38.20 ± 5.29	0.973
Abstinence period (days)	3.70 ± 0.82	3.40 ± 1.17	3.10 ± 0.74	0.362
BMI (kg/m^2^)	27.74 ± 3.38	27.83 ± 4.22	27.43 ± 2.28	0.952
Semen volume (mL)	3.09 ± 0.42	3.09 ± 0.74	3.36 ± 0.79	0.645
Sperm concentration (×10^6^/mL)	52.62 ± 7.97	97.21 ± 9.26	54.98 ± 7.45	<0.001
Total sperm count (×10^6^/ejaculate)	161.89 ± 27.50	303.02 ± 88.32	184.72 ± 50.89	<0.001
Progressive motility (%)	52.55 ± 6.12	51.83 ± 6.33	52.74 ± 6.40	0.946
Total motility (%)	63.68 ± 7.69	62.83 ± 8.55	67.78 ± 5.37	0.232
Normal morphology (%)	6.58 ± 0.94	6.26 ± 0.88	11.21 ± 1.35	<0.001
Vitality (%)	70.74 ± 5.47	68.85 ± 5.27	68.91 ± 4.21	0.672
Smoking, yes/no (number)	3/7	4/6	3/7	0.861
Alcohol, moderate/low-none (number)	3/7	3/7	4/6	0.861

**Table 2:** Small RNA sequencing output and quality metrics across the six libraries.

Sample Name	Total Reads	Clean Reads	Clean-Read Retention (%)	Mapping Rate
NORMAV1	20,430,697	19,488,854	95.39%	80.50%
NORMAV2	20,938,234	20,246,672	96.70%	78.03%
NORMCOUNT1	21,482,577	20,889,061	97.24%	78.36%
NORMCOUNT2	20,785,934	20,041,901	96.42%	76.54%
NORMMOR1	20,475,480	20,105,212	98.19%	73.35%
NORMMOR2	20,890,543	20,456,743	97.92%	75.56%

**Table 3:** Identification of known and novel miRNAs across the six small RNA sequencing libraries.

Sample Name	Known Mature miRNAs	Known Precursors	Novel Mature miRNAs	Novel Precursors
NORMAV1	465	439	12	11
NORMAV2	469	443	12	12
NORMCOUNT1	537	510	15	15
NORMCOUNT2	530	506	14	14
NORMMOR1	476	453	13	13
NORMMOR2	478	454	12	13

**Table 4:** Top 3 most strongly upregulated and downregulated miRNAs in the comparison of NORMCOUNT vs. NORMAV. The table includes information on the direction of change, log_2_ fold changes, and adjusted *p-values* (*q-values*).

miRNAs	log_2_ Fold Change	*q Value*	Dysregulation
hsa-miR-142-3p	−6.90	3.7494 × 10^−21^	Down
novel_75	−6.66	1.4382 × 10^−15^	Down
hsa-miR-145-5p	−6.36	3.8959 × 10^−13^	Down
hsa-miR-891b	7.50	6.4209 × 10^−19^	Up
hsa-miR-34c-3p	7.12	3.5729 × 10^−15^	Up
novel_46	6.32	1.4206 × 10^−9^	Up

**Table 5:** Top 3 most strongly upregulated and downregulated miRNAs in the comparison of NORMAV vs. NORMMOR. The table includes information on the direction of change, log_2_ fold changes, and adjusted *p-values* (*q-values*).

miRNAs	log_2_ Fold Change	*q Value*	Dysregulation
hsa-miR-891b	−6.50	3.1279 × 10^−13^	Down
hsa-miR-34c-3p	−6.24	2.4262 × 10^−11^	Down
hsa-miR-664a-3p	−5.80	9.0546 × 10^−9^	Down
hsa-miR-1-3p	7.15	2.2312 × 10^−15^	Up
novel_75	7.05	2.3315 × 10^−14^	Up
hsa-miR-889-3p	6.66	1.702 × 10^−11^	Up

**Table 6:** Top 3 most strongly upregulated and downregulated miRNAs in the comparison of NORMCOUNT vs. NORMMOR. The table includes information on the direction of change, log_2_ fold changes, and adjusted *p-values* (*q-values*).

miRNAs	log_2_ Fold Change	*q Value*	Dysregulation
hsa-miR-145-5p	−5.23	3.538 × 10^−07^	Down
hsa-miR-23b-5p	−5.04	1.7417 × 10^−06^	Down
hsa-miR-411-5p	−4.91	3.5655 × 10^−07^	Down
hsa-miR-1-3p	5.32	3.1073 × 10^−05^	Up
hsa-miR-106a-3p	5.12	9.8386 × 10^−05^	Up
hsa-miR-708-3p	4.71	0.0007872	Up

## Data Availability

The raw sequencing data generated in this study have been deposited in the NCBI Sequence Read Archive (SRA) under BioProject accession number PRJNA1543036.

## Supplementary Materials

The following supporting information can be downloaded at https://www.mdpi.com/article/10.3390/biom16101514/s1, Table S1: De-identified individual-level demographic, lifestyle, and semen characteristics of the study participants. Two independent semen analyses were performed for each participant. Phenotypic classification was based on consistent fulfillment of the predefined group criteria across both assessments; Table S2: Complete differential expression analysis of sperm miRNAs across the three normozoospermic phenotypic groups; Table S3: Differentially expressed miRNAs shared across all three pairwise comparisons. The table presents the 19 miRNAs identified as significantly differentially expressed in all three comparisons: NORMCOUNT vs. NORMAV, NORMCOUNT vs. NORMMOR, and NORMAV vs. NORMMOR; Table S4: Strongly validated miRNA–target interactions identified for differentially expressed miRNAs in the NORMCOUNT vs. NORMAV comparison according to miRTarget Link 2.0; Table S5: Strongly validated miRNA–target interactions identified for differentially expressed miRNAs in the NORMAV vs. NORMMOR comparison according to miRTarget Link 2.0; Table S6: Strongly validated miRNA–target interactions identified for differentially expressed miRNAs in the NORMCOUNT vs. NORMMOR comparison according to miRTarget Link 2.0; Table S7: Recurrently targeted genes shared across the three pairwise comparisons. Strongly validated miRNA–target interactions were deduplicated at the miRNA–gene pair level. For each shared target gene, the number and identity of differentially expressed miRNAs targeting the gene are reported separately for NORMCOUNT vs. NORMAV, NORMAV vs. NORMMOR, and NORMCOUNT vs. NORMMOR; Figure S1: Significant GO Cellular Component terms associated with the overlap gene targets of DE miRNAs for the NORMCOUNT vs. NORMAV comparison; Figure S2: Significant GO Molecular Function terms associated with the overlap gene targets of DE miRNAs for the NORMCOUNT vs. NORMAV comparison; Figure S3: Significant GO Cellular Component terms associated with the overlap gene targets of DE miRNAs for the NORMAV vs. NORMMOR comparison; Figure S4: Significant GO Molecular Function terms associated with the overlap gene targets of DE miRNAs for the NORMAV vs. NORMMOR comparison; Figure S5: Significant GO Cellular Component terms associated with the overlap gene targets of DE miRNAs for the NORMCOUNT vs. NORMMOR comparison; Figure S6: Significant GO Molecular Function terms associated with the overlap gene targets of DE miRNAs for the NORMCOUNT vs. NORMMOR comparison.

## Abbreviations

The following abbreviations are used in this manuscript:

BP	Biological Process
CC	Cellular Component
DE	Differentially Expressed
GO	Gene Ontology
KEGG	Kyoto Encyclopedia of Genes and Genomes
MF	Molecular Function
NORMAV	Reference normozoospermic men
NORMCOUNT	Normozoospermic men exhibiting markedly elevated sperm concentration
NORMMOR	Normozoospermic men exhibiting a markedly increased proportion of morphologically normal spermatozoa
WHO	World Health Organization
